# A mobile biosafety microanalysis system for infectious agents

**DOI:** 10.1038/srep09505

**Published:** 2015-03-30

**Authors:** Daniel R. Beniac, Shannon L. Hiebert, Christine G. Siemens, Cindi R. Corbett, Tim F. Booth

**Affiliations:** 1 National Microbiology Laboratory, Public Health Agency of Canada, 1015 Arlington Street, Winnipeg, Manitoba. R3E 3R2, Canada; 2 Department of Medical Microbiology, University of Manitoba, Winnipeg, Manitoba. R3E 0W3, Canada

## Abstract

Biological threats posed by pathogens such as Ebola virus must be quickly diagnosed, while protecting the safety of personnel. Scanning electron microscopy and microanalysis requires minimal specimen preparation and can help to identify hazardous agents or substances. Here we report a compact biosafety system for rapid imaging and elemental analysis of specimens, including powders, viruses and bacteria, which is easily transportable to the site of an incident.

Imaging techniques have the advantage that the type of microbe or other threat present in a sample can often be quickly recognized, saving time and allowing specific tests to be selected to more fully identify the agent^1^,^2^. Although immunological detection and nucleic acid amplification capabilities are routinely deployed with mobile laboratories^3^, imaging has the advantage that it can allow novel pathogens to be identified, often to genus, without the need for any prior knowledge of the type of agent present. This information is usually necessary to select specific diagnostic probes (for example antibodies or primer-probe combinations) in conventional tests that detect proteins or nucleic acids. The capability to deploy diagnostic laboratory equipment and personnel closer to the sites of natural outbreaks or potential incidents greatly improves the timeliness of diagnostic testing^3^. The agents of greatest concern include the category A bioterrorism agents anthrax, botulism, plague, smallpox, tularemia, and Ebola and Lassa viruses^4^. Since the anthrax mailings in 2001, which led to 22 infections and five deaths^4^, there have been numerous hoaxes and “suspicious powder” alarms worldwide^5^, and over $50 billion has been spent to counter potential bioterrorist events^6^. More biological containment laboratory capacity has been developed, but these facilities are still rare worldwide^7^. The compact system described here is based on the combination of several well-established, reliable technologies and can provide a cost-effective solution for rapid investigation of biological threats.

A compact scanning electron microscope (SEM) able to give a resolution of 5 nm and accept an X-ray detector for microanalysis, the JEOL JCM-5700, was selected. Although the SEM has a full-size column, its chassis is only 60 cm wide, to fit through most laboratory doorways. All control functions can be remotely controlled electronically from a separate external console. A Class III biosafety cabinet, or “glove box” was designed to house the entire microscope system (Fig. 1, Supplementary Figs. 1–3). The system has motorized external control of most functions allowing the operator to control the movement of the stage, selection of magnification and focusing, and motorized positioning of the detector using controls on the outside of the biosafety enclosure. The cabinet is maintained under negative pressure, with high-efficiency particulate air (HEPA) filtered exhaust and has a specimen pass-through air lock chamber and glove ports to allow specimen changing. The microscope is an air cooled model and does not require liquid nitrogen. After use, the microscope can be decontaminated with a vaporous hydrogen peroxide system^8^. The entire system can be transported to a new location and be operational within 1–2 hours of arrival. Minor servicing and repairs can be carried out while maintaining biological containment.

We tested the equipment and methods for microanalysis using mock specimens of agents that would be important to identify quickly during emergency investigations. For investigation of viruses, we used vaccinia virus as a surrogate for smallpox, and pseudocowpox virus as a typical parapox virus that might need to be identified (to rule out smallpox) during an outbreak of vesicular disease (Fig. 2). As an example of a haemorrhagic fever agent, we used Ebola virus (Fig. 2). Aqueous virus suspensions were filtered through polycarbonate filters that can then be directly observed by SEM. Filtration allows large volumes of dilute pathogens to be concentrated: the detection limit is about 1000 particles per sample and viruses at concentrations of 100 per ml can easily be detected^9^, greatly increasing the likelihood of detection. Virus concentration is thus not a limiting factor for detection with filtration. This method also has the advantage that any debris larger than the pore size is removed, which could otherwise obscure the virus particles. This makes this approach applicable to both samples grown in tissue culture (as presented in this report), as well as any biological fluid from a patient sample. For example we have previously used this method to identify pathogens in urine and blood samples. Pore sizes ranging from 10 nm can catch the smallest viruses, while a pore size of up to 20 μm in diameter is suitable for larger bacteria. Very small virus particles (smaller than 30 nm in diameter) cannot be easily seen by SEM. However, all of the category A agents are above this size range and are thus able to be resolved by SEM.

Our results show that the system was able to distinguish between two families of poxviruses, (Fig. 2). Vaccinia, an orthopox virus, is brick-shaped and larger (approximately 360 × 270 × 250 nm) than pseudocowpox virus (a parapoxvirus) which also has a more cylindrical pill shape (160–190 diameter, 250–300 nm long). Ebola virus was also easily identified in SEM by its distinctive filamentous morphology^10^,^11^ and the presence of comma-shaped virions (Fig. 2).

Spores of *Bacillus cereus* (as a proxy for anthrax) were clearly identifiable using the SEM and high resolution details such as the distinctive exosporium were observed equally as well as with TEM (Fig. 2). Bacterial samples of *Salmonella* and *Listeria monocytogenes* were easily recognizable by their morphology and surface features such as the 20 nm flagellae were also apparent (Supplementary Fig. 4).

All specimens were also observed by negative-stain transmission electron microscopy (TEM)^1^,^12^ for comparison (Fig. 2, Supplementary Fig. 4). SEM has the advantage that the surface textures of the specimen are easily seen, and it can be used to directly observe bulky materials at low magnification. The traditional TEM technique has the disadvantage that it can only be used with dehydrated thin specimens, less than 200 nm thick. Many of the particles in powders are much thicker than this. For example, table salt particles are approximately 5 μm in diameter, more than 2000 times bigger than the thickest sample that can be observed by TEM. Crumbly or volatile specimens also present a problem since they are unstable and can contaminate the ultra-high vacuum of a TEM, but these types of specimen can be well tolerated using the higher specimen chamber pressures at which modern SEMs can operate. The additional specimen preparation steps required for TEM take time, and may also alter the specimen or cause artifacts. Moreover, a TEM is a large piece of equipment that cannot be moved without extensive disassembly, requires stringent stable environmental parameters for operation, and cannot be easily contained in a biosafety enclosure.

Several “white powders” were analysed as examples of the type of specimens that might be encountered during investigation of suspect bioterrorism events. These included table salt, domestic sugar, artificial sweeteners (sodium cyclamate, sucralose), gypsum board, and dried milk powder. (Fig. 2, Supplementary Figs. 5, 6, 7). The SEM images show the distinctive crystalline forms, the presence of fibres, and varied particle sizes of the different powders. The X-ray spectra demonstrate the relative elemental abundance profiles in the specimen (Fig. 2, Supplementary Fig. 5), and elemental images show the location of the specific elements within a sample (Supplementary Fig. 7), both of which are useful as a signature for forensic identification of the substance. Toxic heavy metals and other elements of high atomic weight are readily apparent in X-ray spectra. For example, the domestic sugar sample contains traces of calcium, silicon, sodium, magnesium, sulphur and aluminum from impurities or additives that are readily detectable (Fig. 2).

The X-ray spectra and elemental maps were collected using the X-max 80 mm^2^ silicon drift detector (SDD) with the INCA EnergySEM 350 microanalysis software package (Oxford Instruments, Abingdon, UK). This system has 2048 channels with an energy resolution of 129 eV at Mn Kα, with a light element sensitivity that can detect beryllium. The system is capable of collecting spectra, elemental images, and quantitating the relative elemental composition in a specimen. The operation of this system in the SEM enclosure does not affect the energy resolution, or any of the other operations of the system as per the manufacturer's specifications. Some elements generate X-rays with overlapping peak positions (by both energy and wavelength) that are difficult to separate. Thus sensitivity varies according to the elemental composition of the specimen. In general, a useful X-ray detection limit is 0.1%, but can be as low as 0.01% for elements of large atomic mass against a low atomic mass background, or where the X-ray spectral peaks are well separated^13^. In general, substances such as toxic heavy metals added to an otherwise harmless substance (such as foods- which contain mostly elements of low atomic mass) can often be easy to detect with the technique.

The combination of X-ray microanalysis and SEM all within the same enclosure provides a safe and powerful forensic tool. The SEM images permit the morphological identification of a microorganism, and the X-ray microanalysis can give information on the elemental composition of both the organic and inorganic components. For example, elemental composition data such as a high calcium concentration, can be used to differentiate bacterial spores from other suspicious particles with a similar morphology^14^. In investigating a suspect anthrax powder, its morpohological characteristsics, such as having a high spore concentration, a uniform particle size, and the presence of anti-clumping agents might indicate a material that was deliberately prepared^16^. Thus, a full forensic analysis can be carried out, using a combination of nucleic acid amplification methods to identify the specific strain of agent present, along with microanalysis to determine the particle size, morphology, and elemental composition to help investigate how the material was formulated.

In conclusion, we describe the first electron microscope with elemental microanalysis within a compact class III biosafety cabinet that can be operated externally, while the system is biologically sealed. Along with novel methodology for specimen preparation that we have recently developed^9^, this system is ideal for microscopy and elemental microanalysis of biohazardous specimens. This has been achieved in a highly compact platform that can be easily moved from one room to another. For example, it could be temporarily operated within a biosafety level 3 laboratory, to further increase safety. The capability for operation at the site of incidents can help to avoid delays in transporting specimens to specialized high containment laboratories. For this type of field operation, it is envisaged that the micoranalysis system would form part of a mobile laboratory setup including personal protective equipment and a portable negative air pressure isolation unit for collecting and processing samples^3^,^15^.

## Methods

### Design of the cabinet

**Figure 1:**
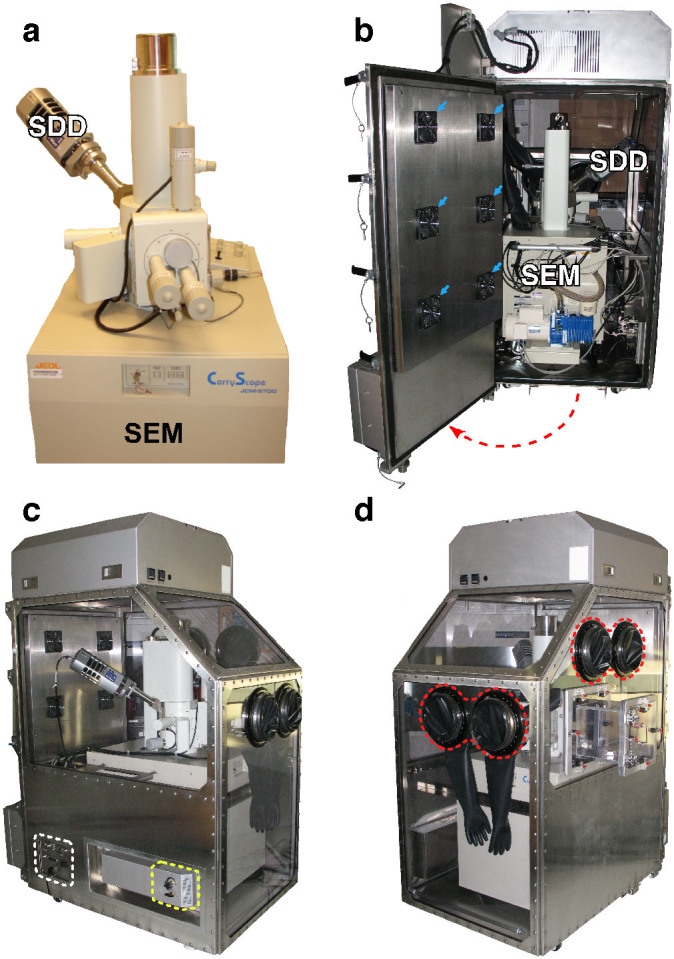
The microscope biosafety enclosure. (a) The microscope is a compact model, although the Oxford X-max 80 mm^2^ silicon drift detector (which needs to be able to move inwards and outwards from the microscope column) increases the width of the system. (b) The SEM enclosure shown with the rear door open. The SEM is moved into the enclosure through this door on wheels (the red dotted line shows the direction of opening). The six Peltier cooler units (blue arrows) and the majority of the system electronics are mounted on the door. (c, d) The enclosure sealed shut with the SEM inside. The HEPA filter air inlet is shown with a yellow dotted line, the electrical bulkhead is identified in white, and the glove ports are highlighted in red.

The best solution was to enclose the entire microscope in the class III biosafety high-efficiency particulate air (HEPA) filtered cabinet maintained at an internal air pressure that is negative to that of the surrounding room (Fig. 1, Supplementary Fig. 1). This avoided problems with designs that contained only part of the microscope system (which would cause difficulties with sealing the joints around complicated parts of the system). Furthermore, the vacuum exhaust is within the containment enclosure, to avoid any potential aerosol hazards that could be created when the microscope chamber is pumped down. A full scale mock-up of the SEM enclosure design was constructed around the JEOL JCM-5700 (Supplementary Fig. 2). The SEM was given a full imaging and spectrum collection operation trial with the mock-up in place. This allowed adjustments to the design to be made before final manufacturing. Operation using the electrical bulkhead was tested to ensure correct electronic function, and adjustments were made to allow ergonomic positioning of the glove ports and pass through box for specimen exchange, aperture alignment and filament replacement.

The cabinet is equipped with three HEPA filters for air intake, exhaust, and the pass-through chamber which acts as an airlock for bringing infectious specimens into the microscope (Fig. 1c, Supplementary Figs. 1f, 3c). The filters can easily be removed while preventing any external contamination (known as “bag in/bag out” replacement). Ports are available for decontaminating the entire equipment after use, using vaporous hydrogen peroxide (Supplementary Fig. 3d). Sensors provide a read out of the temperature and pressure inside the enclosure, which is maintained at a lower level than outside, so that when the pass-through chamber is opened to insert specimens, air is drawn inwards, protecting the outside environment from contamination (Supplementary Fig. 3a). Enclosing the equipment in an air-tight box created a problem with the air cooling of the microscope, which is designed to work in a laboratory room with adequate ventilation and temperature control systems. Six Peltier refrigerator units were included to provide sufficient cooling (Fig. 1b, Supplementary Fig. 1f, blue arrows).

The design also has a rear door through which the microscope can be wheeled out on ramps for servicing (Fig. 1b, Supplementary Fig. 3b). There is external control of all of the microscope's controls including motorized stage movement, magnification selection, beam intensity and focussing functions. The electronics for external control are connected via an electrical bulkhead (Fig. 1c, Supplementary Fig. 3e). A general purpose port (blue circle, in Supplementary Fig. 3e) was included for installation of additional detectors or accessories if required.

### Wet sample preparation for SEM

All fluid samples were filtered using 13 mm diameter SPI–pore polycarbonate track etch filters (SPI supplies, West Chester Pennsylvania, USA), held in 13 mm Swinnex® filter holders (Millipore, Billerica, Massachusetts, USA) attached to syringes with Luer-Lok® couplings to prevent sample leaks. In a Class II Biosafety Cabinet, bacterial suspensions were made form growth on agar plates. Approximately two loop-fulls of bacteria were suspended in 1 ml PBS. If the suspension was too turbid a 10X dilution was made. The filter was first wetted by passing 2 ml of PBS through the apparatus. Then 0.2 ml of the bacterial suspension was applied to the filter with a 1 ml syringe, followed by three consecutive 2 ml washes of PBS using a 2 ml syringe. Finally 2 ml of 4% glutaraldehyde was applied with a 2 ml syringe. After one hour wait, the filter was washed with 2 ml 50% ethanol, 2 ml 70% ethanol, 2 ml 85% ethanol, 2 ml 95% ethanol, and then 2 ml of 100% ethanol and finally air dried. Syringes were either operated by hand, or with a Legato 200 syringe pump (KD Scientific, Holliston, Massachusetts, USA).

### Gold coating

Filters were cut and mounted on an SEM stub using double-sided adhesive carbon disc and silver flash paint to create a contact between the stub and the filter paper. The samples were sputtered with gold using a Quorum Q150R S (Quorum Technologies, East Sussex, UK) containing a 0.1 mm gold target. The sample was pumped down, purged with argon and sputtered with gold for 120 sec while on a rotating stage.

### Dry sample preparation for SEM

Working in a class II biosafety cabinet, powder samples were directly mounted onto double-sided adhesive carbon discs attached to metal specimen stubs, using a spatula to sprinkle small quantities. The powder was then gently pressed with the spatula to improve adherence. The stub was then inverted over a waste container and tapped to remove any excess loose particles.

### Operation of the SEM in the biosafety enclosure

When the enclosure is turned on the negative pressure alarm sounds until the enclosure reaches the operational 0.5″ H_2_O below ambient pressure which is usually achieved in less than one minute. This confirms both operational status of the system as well as the functioning of the alarm system. The negative pressure inside is indicated in red on the controller display (Supplementary Fig. 3a). Temperature control is automatic and maintains an internal temperature of 20.5°C. The SEM (JEOL CarryScope, JEOL Ltd., Tokyo, Japan) is computer controlled, and equipped with motor drives for X, Y and Z motion which are connected to the electrical bulk head of the enclosure (Supplementary Fig. 3e). The only differences in the operation of the SEM is that any time the SEM has to be physically touched the glove ports or specimen pass through must be used (Fig. 1d, Supplementary Fig. 1e). Therefore operations including turning on the SEM, opening and the closing stage for specimen insertion, specimen stage tilting, in-plane rotation of specimen in stage, alignment of condenser aperture, servicing of the electron source, and condenser aperture adjustment all require use of the glove ports. All other features of the SEM are electronically controlled externally (shift, focus, magnification, stigmatism, contrast, and brightness). The SEM was operated at 4 kV, with a 7 mm working distance, and with a 30 μm condenser aperture. Images (2560 × 1920 pixels) were collected using the secondary electron detector with an acquisition time of 160 seconds.

### X-ray microanalysis

X-ray spectra were collected using the X-max 80 mm^2^ silicon drift detector (SDD) with the INCA microanalysis software package (Oxford Instruments, Abingdon, UK). The entire detector is electronically controlled externally and is connected to the computer through the electrical bulkhead of the biosafety enclosure (Supplementary Fig. 3e). The X-max SDD is cooled by a Peltier cooler, and so does not require liquid nitrogen. For X-ray microanalysis the SEM was operated at 20 kV, with a 10 mm working distance, and 100 μm condenser aperture. Maps and line scans were collected at 512 × 352 pixels with 2048 X-ray channels and 50 frames per acquisition with a dwell time of 100 μs per pixel: each acquisition took 15 minutes.

### Virus cultures

Zaire Ebola virus was propagated in Vero E6 cells and prepared as previously described^17^. Samples were analysed by SDS-Page and Western blotting, and rendered non-infectious by fixation with 4% paraformaldehyde. Excess fixative was removed by placing the fixed samples in a Slide-A-Lyzer G2 cassette with a 0.5 ml capacity, and a 10,000 MWCO (Thermo Scientific Pierce Protein Research Products, Rockford, Illinois, USA), followed by dialysis against PBS. All work with infectious Ebola virus (virus culture and purification) was performed in the biosafety level 4 laboratories at the National Microbiology Laboratory of the Public Health Agency of Canada, Winnipeg, Manitoba.

Baby hamster kidney fibroblast cells (BHK-21: ATCC) were grown in Dulbecco's Modified Eagle's Medium (Gibco) containing 10%fetal bovine serum (FBS) (Gibco) and 1X penicillin/streptomycin/L-glutamine (Gibco). BHK-21 cells were inoculated with Modified Vaccinia Ankara (MVA) (kindly provided by Dr. Jingxin Cao, National Microbiology Laboratory) for 1 hour at 37°C. After washing with PBS, complete growth medium was added and the cells incubated at 37°C for 48 hours. MVA was harvested by freeze-thawing cell cultures 3X, alternating between -80°C and room temperature. Following final thawing, the supernatant was clarified by centrifugation at 3000 × g for 3 minutes to remove the cell debris.

### Bacterial cell cultures

*Bacillus cereus* was cultured on CAB plates, and incubated at 37°C for 24 hours. 300 ml of 1/10 Columbia broth containing 0.1 mM MnSO_4_, were inoculated with a loopful of *Bacillus cereus*, and incubated at 37°C for 96 hours on an orbital shaker. The culture was centrifuged at 4900 g for 15 minutes at 4°C. The supernatant was decanted and the pellet was washed three times in sterile deionized water, followed by centrifugation at 4900 × g for 15 minutes at 4°C. The entire spore preparation was resuspended in 40 ml of ethanol, and transferred to a 50 ml tube, then incubated at room temperature for 2 hours. Then it was centrifuged at 4900 g for 15 minutes at 4°C, and the ethanol was decanted off. This was followed by two washes with 40 ml of sterile deionized water, and centrifuging at 4900 g for 15 minutes at 4°C. The remaining wash was decanted off. The final spore pellet was resuspended in 10 ml of sterile deionized water. Non-Typhi *Salmonella* was grown overnight on a semi-solid agar plate at 35°C. *Listeria monocytogenes* was grown in tryptose phosphate agar (TPA) motility tubes at room temperature for two days, and then sub-cultured on to a TPA plate at room temperature for an additional two days.

### Sample preparation for TEM

Fluid samples for transmission electron microscopy were fixed in 2% glutaraldehyde/1% paraformaldehyde. Samples were adsorbed to glow discharged carbon-coated formvar films on a 400-mesh copper grids for 1 min, and negatively stained with 2% methylamine tungstate (Nano- W; Nanoprobes, Yaphank, NY, USA). Specimens were observed at 200 kV in an FEI Tecnai 20 transmission electron microscope (FEI Company, Hillsboro, OR, USA) operated, and at instrument magnifications of X25,500 to X71,000. Digital images of the specimens were acquired using an AMT Advantage XR 12 CCD camera (AMT, Danvers, MA, USA).

## Author Contributions

T.F.B. and D.R.B. designed the enclosure, and conceived the experiments. D.R.B., C.G.S. and S.L.H. conducted the experiments. C.R.C. contributed to the bacterial spore experiments. T.F.B. and D.R.B. wrote the manuscript.

## Supplementary Material

Supplementary Information Supplementary information

## Figures and Tables

**Figure 2:**
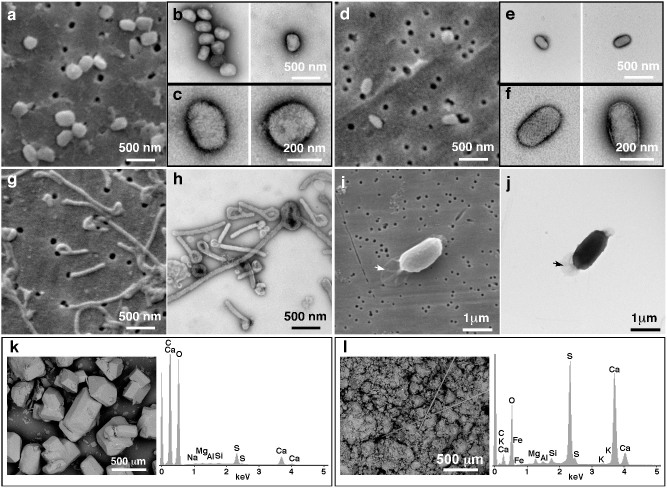
Microscopy and microanalysis of pathogens and mock-bioterrorist agents. Images were acquired using both SEM (a, d, g, i, k, l), and TEM are shown for comparison (b, c, e, f, h, j). Vaccinia virus (a–c), Pseudocowpox virus (d–f), Ebola virus (g, h) and *Bacillus cereus* (i, j) are shown. The arrows in (i, j) indicate the exosporium. SEM image and X-ray spectra of domestic sugar (k), and crushed drywall gypsum board (l), as samples of mock-bioterrorism “white powder” agents.
