# Supplementary material for: A mobile biosafety microanalysis system for infectious agents
Source: Sci Rep. 2015 Mar 30;5:9505. doi: 10.1038/srep09505 (PMC4377622; doi:10.1038/srep09505)
Supplement: Supplementary Information [file srep09505-s1.pdf]

Supplemental Information

A mobile biosafety microanalysis system for infectious agents

Daniel R. Beniac[1], Shannon L. Hiebert[1], Christine G. Siemens[1], Cindi R. Corbett[1,2], and Tim F. Booth[1,2]*

1 **National Microbiology Laboratory, Public Health Agency of Canada, 1015 Arlington Street, Winnipeg, Manitoba, R3E 3R2, Canada.**
2 **Department of Medical Microbiology, University of Manitoba, Winnipeg, Manitoba, R3E 0W3, Canada.**

***E-mail: tim.booth@phac-aspc.gc.ca**

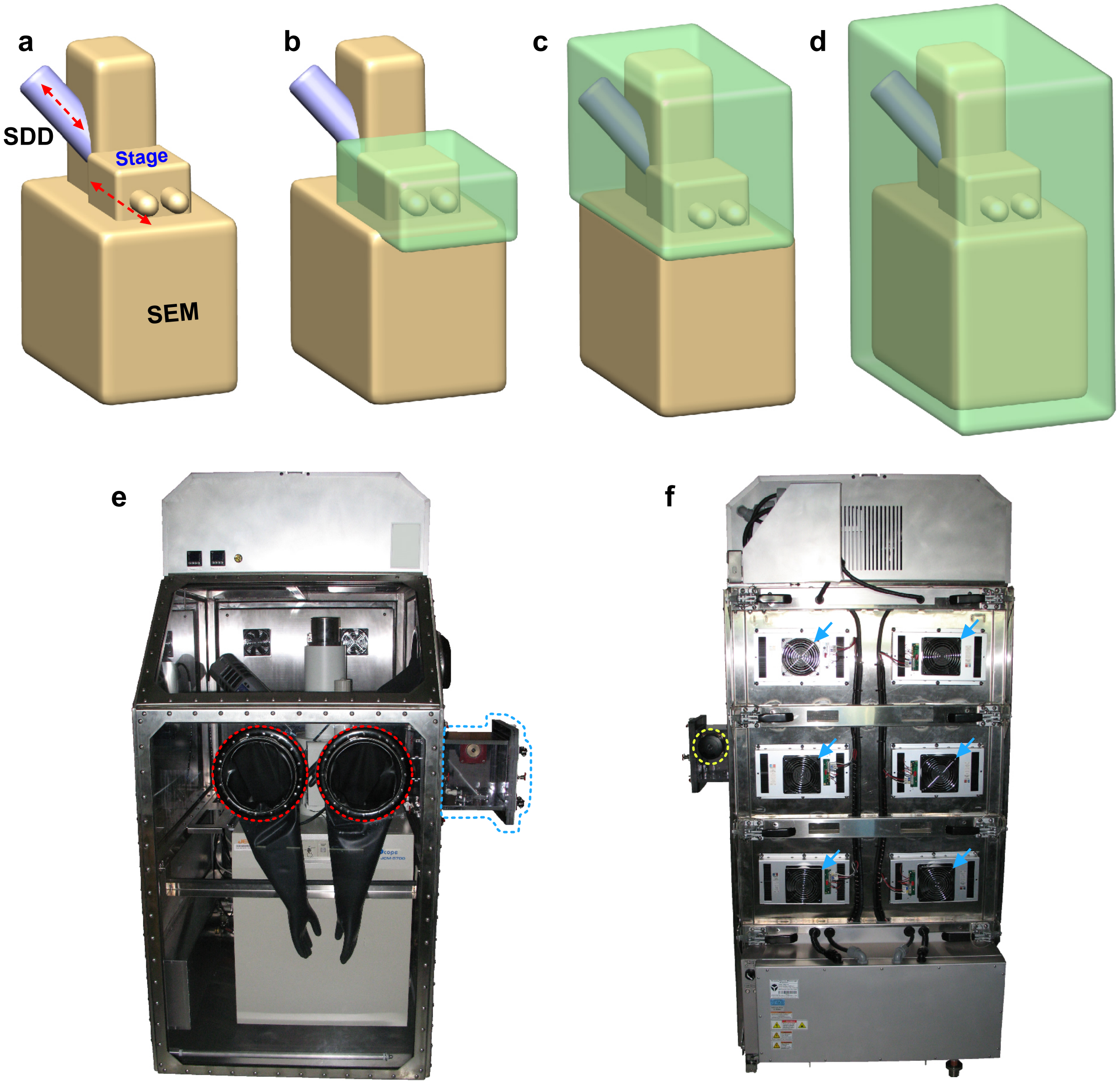

Supplemental Figure 1 | Design of the SEM enclosure. (**a**) Computer drawing of the scanning electron microscope (SEM) equipped with silicon drift detector (SDD) for X-ray microanalysis. The red arrows indicate the movements. The three alternative enclosure designs are (**b**) stage only, (**c**) top portion of microscope including optical column, and (**d**) entire microscope. The prototype enclosure shown from two perspectives (**e**,**f**). The specimen pass-through chamber is indicated by a blue dashed outline, and glove ports are highlighted in red (**e**). The HEPA filter on the pass through chamber is circled in yellow, and the six Peltier coolers are indicated by blue arrows (**f**).

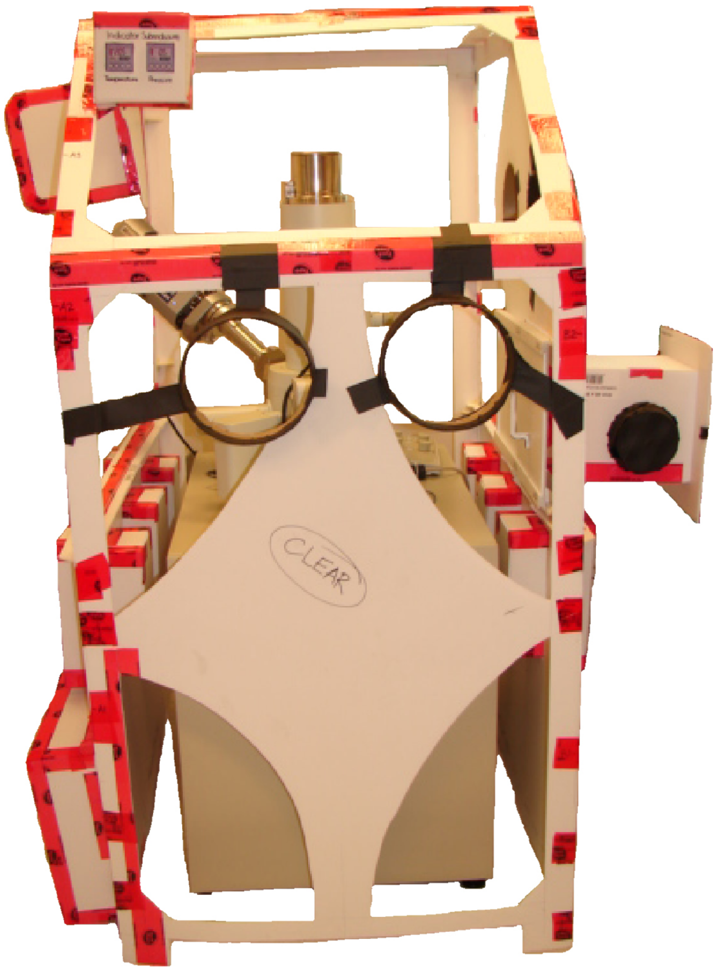

Supplemental Figure 2 | A full operation trial of the microscope with a mock-up of the enclosure in place was undertaken. This allowed adjustments of the design to be made before final manufacturing. Testing included: operation of the electrical bulkhead to ensure correct function, making sure that the glove ports were ergonomically positioned to allow easy specimen exchange, aperture alignment, and filament replacement.

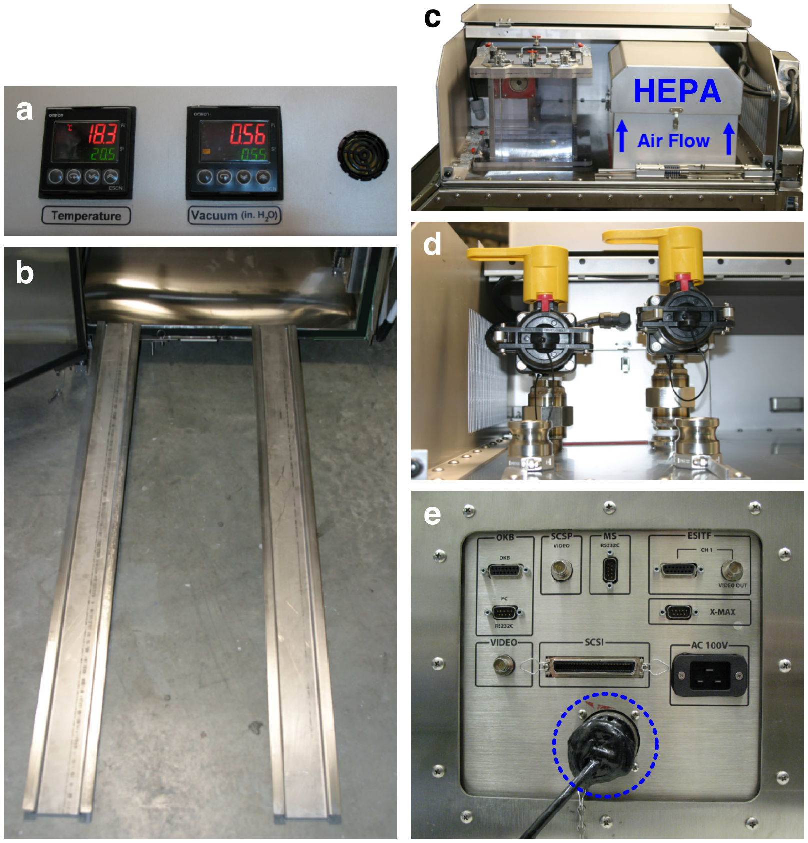

Supplemental Figure 3 | Features of the final manufactured SEM enclosure. Close-up images of the temperature and pressure gauges (**a**), and the 150 cm ramp (**b**) are shown. The HEPA filter is shown with the direction of air flow in blue (**c**), the vaporous hydrogen peroxide ports (**d**: yellow), and the electrical bulkhead (**e**). The general purpose port is indicated by the dashed blue circle (**e**).

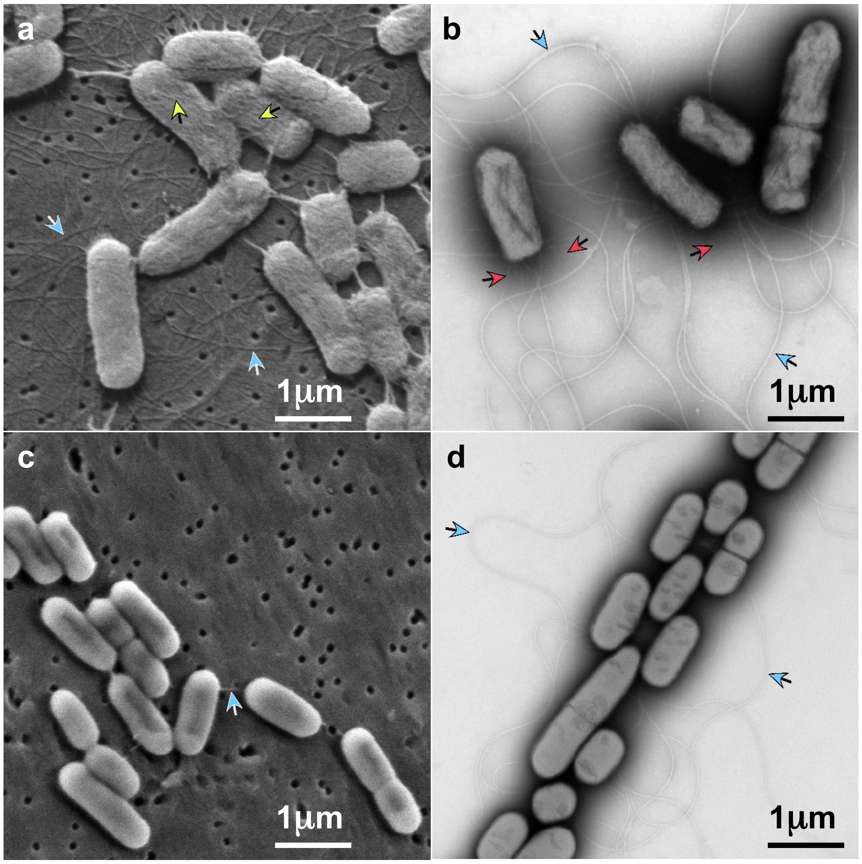

Supplemental Figure 4 | Additional images of biological samples. Images were acquired using both SEM (**a,c**), and TEM (**b,d**) . Specimens were *Salmonella* (non-Typhi) (**a,b**), *Listeria monocytogenes* (**c,d**). Color code for arrows: flagellae, (blue arrows); fimbriae on the substrate, (red); fimbriae on the surface of bacterial cells, (yellow).

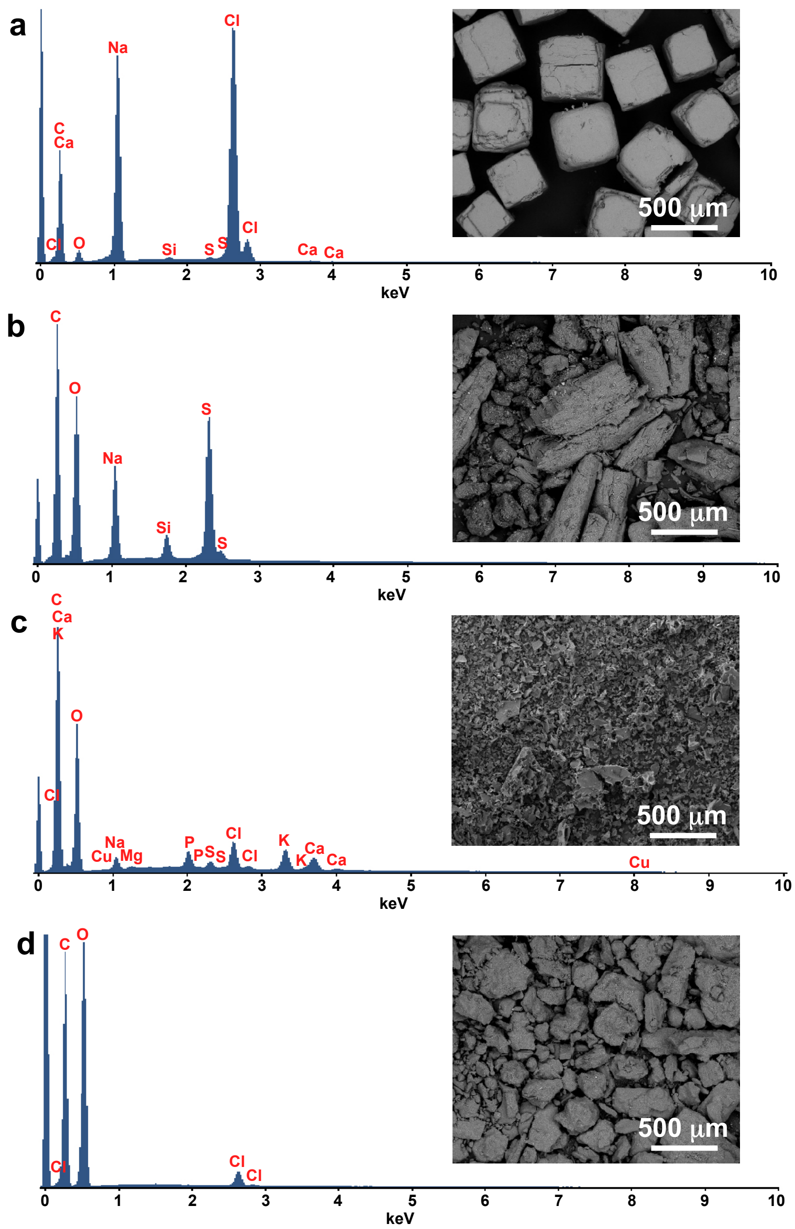

Supplemental Figure 5 | SEM images and X-ray spectra of mock-bioterrorist agents. The four "white powder" agents shown are (**a**) table salt, (**b**) sodium cyclamate based artificial sweetener, (**c**) lyophilized milk, and (**d**) sucralose based artificial sweetener. SEM images are presented at the same magnification to show the differences in particle size and shape of the samples.

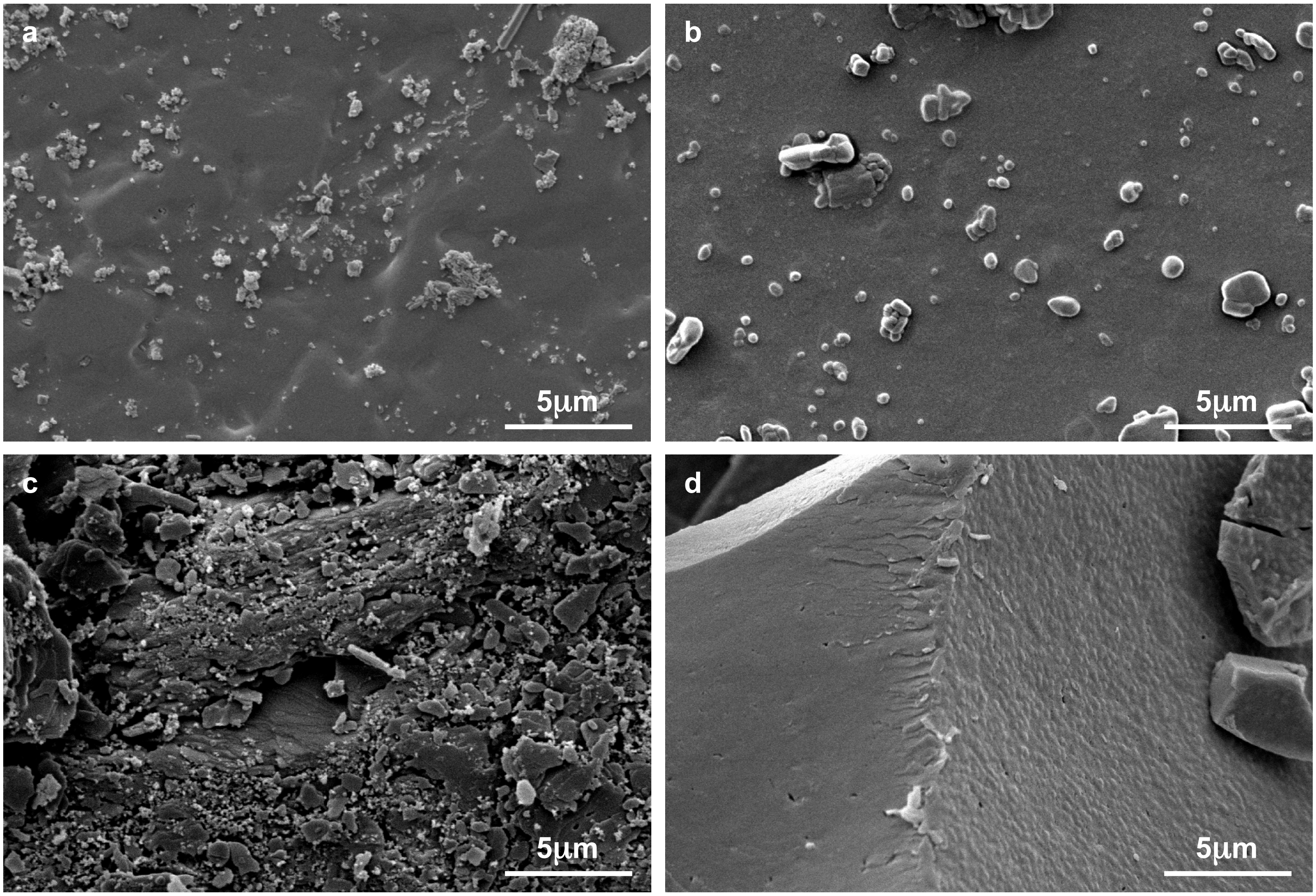

Supplemental Figure 6 | Higher magnification SEM images of “white powder” mock-bioterrorist agents; (**a**) table salt, (**b**) sugar, (**c**) sodium cyclamate based artificial sweetener, and (**d**) lyophilized milk.

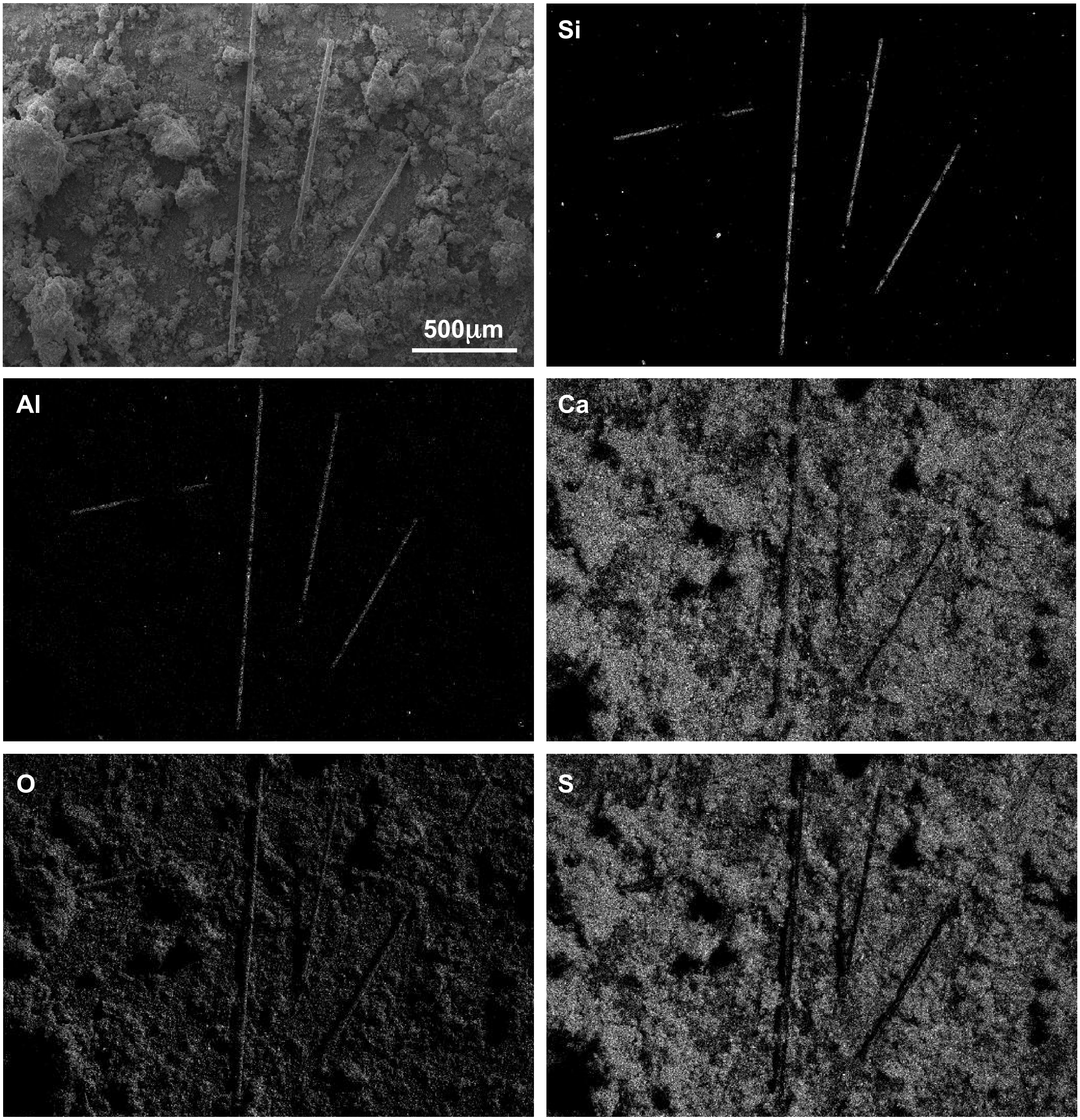

Supplemental Figure 7 | SEM image of crushed gypsum board (top left), and corresponding elemental images. Gypsum board (also known as plasterboard, wallboard, or drywall) is composed of dihydrous calcium sulfate ($CaSO_4 \cdot 2H_2O$). These elements give rise to the strong Ca, O, and S maps, whereas the glass fibers in the board give rise to the Si and Al signals.
